# A Novel S100 Family-Based Signature Associated with Prognosis and Immune Microenvironment in Glioma

**DOI:** 10.1155/2021/3586589

**Published:** 2021-09-29

**Authors:** Yifang Hu, Jiahang Song, Zhen Wang, Jingbao Kan, Yaoqi Ge, Dan Wang, Rihua Zhang, Wensong Zhang, Yun Liu

**Affiliations:** ^1^Department of Geriatrics, The First Affiliated Hospital of Nanjing Medical University, Nanjing, Jiangsu, China; ^2^Department of Radiation Oncology, The First Affiliated Hospital of Nanjing Medical University, Nanjing, Jiangsu, China; ^3^Department of Neurosurgery, The Affiliated Brain Hospital with Nanjing Medical University, Fourth Clinical College of Nanjing Medical University, Nanjing, Jiangsu, China; ^4^Department of Pharmacy, The First Affiliated Hospital of Nanjing Medical University, Nanjing, Jiangsu, China; ^5^Department of Medical Informatics, School of Biomedical Engineering and Informatics, Nanjing Medical University, Nanjing, Jiangsu, China

## Abstract

**Background:**

Glioma is the most common central nervous system (CNS) cancer with a short survival period and a poor prognosis. The S100 family gene, comprising 25 members, relates to diverse biological processes of human malignancies. Nonetheless, the significance of S100 genes in predicting the prognosis of glioma remains largely unclear. We aimed to build an S100 family-based signature for glioma prognosis.

**Methods:**

We downloaded 665 and 313 glioma patients, respectively, from The Cancer Genome Atlas (TCGA) and Chinese Glioma Genome Atlas (CGGA) database with RNAseq data and clinical information. This study established a prognostic signature based on the S100 family genes through multivariate COX and LASSO regression. The Kaplan–Meier curve was plotted to compare overall survival (OS) among groups, whereas Receiver Operating Characteristic (ROC) analysis was performed to evaluate model accuracy. A representative gene S100B was further verified by in vitro experiments.

**Results:**

An S100 family-based signature comprising 5 genes was constructed to predict the glioma that stratified TCGA-derived cases as a low- or high-risk group, whereas the significance of prognosis was verified based on CGGA-derived cases. Kaplan–Meier analysis revealed that the high-risk group was associated with the dismal prognosis. Furthermore, the S100 family-based signature was proved to be closely related to immune microenvironment. In vitro analysis showed S100B gene in the signature promoted glioblastoma (GBM) cell proliferation and migration.

**Conclusions:**

We constructed and verified a novel S100 family-based signature associated with tumor immune microenvironment (TIME), which may shed novel light on the glioma diagnosis and treatment.

## 1. Introduction

Glioma is the most common type of human primary brain cancer, which accounts for approximately 30% of all brain cancer occurrences [1]. Glioma can be divided into low (I-II) or high (III-IV) grades based on the World Health Organization (WHO) criteria. It is difficult to entirely remove tumor tissue during surgery due to the high invasion, infinite proliferation, diffuse infiltration and lack of a clearly boundary of high-grade glioma [2]. Despite advances in surgery, chemotherapy and radiotherapy, glioma is still associated with a poor prognosis, and its median survival is as short as <15 months [3]. However, these therapeutic strategies are limited by drug resistance and tumor recurrence, which are influenced by a complicated gene regulatory network. Therefore, identification of reliable targets and prognostic biomarkers for glioma is urgently required.

The S100 family is a category of low-molecular-weight (10–14 kda), acidic, calcium-binding protein with an EF-hand motif that was first identified from the bovine brain in 1965 by Blake W. Moore [4]. Currently, 25 family members have been described, 16 are clustered together on chromosome 1q21, a locus susceptible to genomic rearrangements in malignant tumors [5]. S100 proteins participate in regulating some cell processes, like proliferation, differentiation, apoptosis, and immune responses. It has also become evident that many specific S100 genes are abnormally expressed in some human tumors, facilitating cancer genesis and development [6]. S100A4, S100B, and S100P, for example, inhibit the phosphorylation of p53 and subsequently attenuate the tumor-suppressive ability of p53 [7, 8]. S100A8/S100A9 activates the MAPK pathway to promote the proliferation of breast cancer (BC) [9]. Increased S100A11 expression has been observed in lung cancer, which activates Wnt/*β*-catenin pathways to facilitate the development of drug resistance and cancer metastasis [10]. Additionally, a number of S100 proteins could be identified as molecular biomarkers to diagnose or predict a specific cancer [11].

This study focused on developing a prognostic nomogram based on the S100 family members to explore the clinical significance of this family for glioma prognosis. The prognostic values and expression profiles of the S100 family in glioma samples were comprehensively evaluated using public resources and bioinformatics analysis. We identified five signature-related genes that are associated with the survival of glioma patients; besides, multiple tumor-related pathways were enriched into the high-risk group. Our results indicate that S100 family-based signature may play a critical role in glioma progression and could be considered as prognostic markers and therapeutic targets for glioma in the future.

## 2. Materials and Methods

### 2.1. Acquisition of Glioma Datasets

Clinical and FPKM RNAseq data of 703 glioma cases were obtained from TCGA database (https://portal.gdc.cancer.gov/) into the training set. Similarly, we also obtained 325 cases from the CGGA database (http://www.cgga.org.cn) into the validation set. According to patient ID, this study compared clinical features of patients with corresponding transcriptome data. Samples were removed if the data did not match. A total of 665 and 313 patients with complete clinical data were finally selected from TCGA and CGGA database for the next analysis, respectively.

### 2.2. Construction and Verification of a Risk Score Prognostic Model Based on S100 Gene Family Members

A Cox proportional hazard regression model was constructed to estimate the prognosis of glioma cases in the TCGA training set. Furthermore, the model's prognostic performance was validated in the CGGA validation set. Firstly, the candidate S100 family genes related to prognosis were identified by univariate Cox regression through “survival” package upon the threshold of *P* < 0.05 [12]. Secondly, overfitting genes were removed through LASSO regression via R package “glmnet” function [13]. Thirdly, R package “glmnet” function was also utilized to build a prognosis prediction nomogram by multivariate Cox proportional hazard regression [14]. The final risk score prognostic model was established with the following formula:

(1) $$\begin{array}{c} \text{risk score}=\sum_{j=1}^{n}\left({\text{Coef}}_{j}\times X_{j}\right). \end{array}$$

Here, Coef_*j*_ stands for coefficient of multivariate Cox regression for gene *j*; n represents the overall hub gene number; *X*_*j*_ indicates relative gene *j* expression within the model.

For exploring the significance of the risk score model in predicting prognosis, glioma cases were classified as a low- or high-risk group according to median risk score value, with high-risk group having a poor prognostic outcome. Then Kaplan–Meier curves were plotted to analyze the OS of two groups of glioma patients through the log-rank test. The sensitivity and specificity of the constructed nomogram were assessed through determining 1-, 3-, and 5-year area under the time-dependent ROC curve (AUC) values using the survival ROC R package, with an AUC > 0.70 denoting a good predictive value.

### 2.3. Integration of Protein-Protein Interaction (PPI) Network and Identification of Hub Genes

This study constructed the protein-protein interaction (PPI) network using the STRING database (http://www.string-db.org/). Cytoscape (https://cytoscape.org/) is often used for visualizing the complicated network and integrating them with attribute data. In the present work, Cytoscape was utilized for building the PPI network and for analyzing the relationships among S100 family members. Following that, the Maximal Clique Centrality (MCC) algorithm in the Cytoscape software (v 3.7.0) was employed to identify hub genes.

### 2.4. Construction and Evaluation of the Nomogram

To provide an approach for quantitatively analyzing the OS of glioma, we used the “rms” R packages to construct a nomogram based on clinical variables and the prognosis signature. A calibration curve [15] was plotted to evaluate the nomogram prediction performance by analyzing the consistency of predicted values with actual measurements.

### 2.5. Gene Set Enrichment Analysis (GSEA)

The Hallmark gene set collections (h.all.v7.2) were downloaded in Molecular Signatures Database (MSigDB) v7.1 (https://www.gsea-msigdb.org/gsea/downloads.jsp). The present work carried out GSEA for comparing the biological functions and pathways related to signature-related genes between low- and high-risk groups from both TCGA and CGGA data sets by the use of GSEA v4.0.3 (https://www.gsea-msigdb.org/). In line with GSEA User Guide, the significant gene sets were selected at the thresholds of FDR *q* < 0.25, NOM *p* < 0.05, and |NES| > 1.

### 2.6. Assessment of Immune Cell Type Fractions

The abundance of immune cell type fraction between low- and high-risk score groups was estimated by CIBERSORT (https://cibersort.stanford.edu/) [16]. CIBERSORT is a new approach that extensively adopted to characterize cellular components in composite tissues based on gene expression profiling data within cancers, and it can obtain ground truth estimates with high consistency in diverse cancer types. LM22, a white blood cell (WBC) gene signature matrix involving 547 genes, was adopted for distinguishing 22 kinds of tumor-infiltrating immune cells (TIICs), such as regulatory *T* cells (Tregs), *T* cells, *B* cells, natural killer (NK) cells, mast cells, dendritic cells (DCs), monocytes, neutrophils, eosinophils, and macrophages.

### 2.7. Cell Culture and Transfection

GBM cell lines U251 and T98G were incubated in Dulbecco's modified Eagle's medium (DMEM) with 10% fetal bovine serum (FBS) at 37°C in a 5% CO_2_ incubator. The logarithmic cells were selected for next experiments. S100B knockdown was achieved by transfecting cells with S100B-siRNA using Lipofectamine® 3000 transfection reagent (Invitrogen, USA) according to the manufacturer's protocol. The gene encoding human S100B was inserted into a plasmid vector (Genechem Incorporation, China). The target sequences for siS100B-1 and siS100B‐2 were 5′-GAGACGGUCAUGCAAGAAATT-3′ and 5′-GAGACGGUCAUGCAAGAAATT-3′, respectively.

### 2.8. RNA Extraction and qRT-PCR

Total RNA was extracted using Trizol reagent (Invitrogen, America), and cDNAs were synthesized using HiScript Synthesis kit (Vazyme, China). Quantitative real-time PCR (qRT-PCR) was then conducted on a StepOnePlus Real-Time PCR system (Applied Biosystems, CA, US) using the Fast SYBR Green Master Mix (Roche, America). Primer sequences in this study are detailed in Table S1.

### 2.9. Western Blotting

Cell protein was extracted using RIPA lysis buffer (P0013D, Beyotime, China). Equal amounts of protein samples were separated on 12.5% SDS-PAGE gels, which were then electrotransferred onto nitrocellulose (NC) membranes (Pall Corporation, USA). The membranes were blocked for 2 h at room temperature with 5% nonfat milk, then incubated overnight at 4°C with a primary antibody (against S100B, Abcam), followed by the corresponding secondary antibody for 2 h.

### 2.10. CCK-8 and EdU Proliferation Assay

Cell counting kit-8 (CCK-8) and 5‐ethynyl‐20‐deoxyuridine (EdU) assays were conducted to evaluate the cells' proliferative ability. Cells were seeded in 96-well plates at a density of 2000 cells per well overnight, and cell growth was assayed at different periods utilizing CCK-8 kit (C0038, Beyotime, China) based on the instruction manual. Absorbance at 450 nm was determined by enzyme labeling (Thermo Scientific Multiskan FC, USA). The EdU test was carried out using a Cell-Light EdU Apollo 567 In Vitro Imaging Kit (Ribobio, China) following the manufacturer's protocol. Cells were first stained with 50 *μ*M EdU for 2 hours, then fixed with 4% paraformaldehyde and permeabilizated with 0.5% Triton X‐100. After three washes with PBS, cells were incubated with 1 × Apollo® for 30 min, followed by DAPI staining. The EdU-positive cells were eventually viewed by fluorescence microscopy (Olympus, Japan).

### 2.11. Transwell Invasion Assay

The Transwell invasion test was carried out in the 24-well Transwell chambers (Corning, USA) precoated with Matrigel (BD Biosciences, USA). The top chambers were seeded with about 5 × 10^4^ cells in serum-free DMEM media, whereas the bottom chambers were filled with DMEM containing 10% FBS. After 24 h of incubation, the penetrated cells were fixed with 4% methanol, then stained with 0.1% crystal violet. Treated cells in each well were finally photographed at random and counted under an inverted microscope (Nikon, Japan).

### 2.12. Data Analysis

GraphPad Prism 5.0 (GraphPad Software, Inc., San Diego, CA, USA) or R software (version 4.0.3) was utilized for statistical analysis. Differences between two groups were compared by Mann–Whitney *U* tests or Students *T*-tests, whereas those across numerous groups were compared by Kruskal–Wallis H tests or one-way ANOVA. Associations of clinical features with risk scores were analyzed by Fisher's exact test and chi-square test. Survival data were analyzed by Kaplan–Meier analysis. The influence of risk score on OS was evaluated through univariate as well as multivariate Cox regression. The prognosis prediction performance of the risk model was assessed through ROC curve analysis. Each experiment was repeated thrice, and results were expressed as mean ± SD. ^*∗*^*P* < 0.05, ^∗∗^*P* < 0.01, and ^∗∗∗^*P* < 0.001 were considered significant.

## 3. Results

### 3.1. Construction of the S100 Family-Based Signature of Glioma in the TCGA Cohort

**Figure 1:**
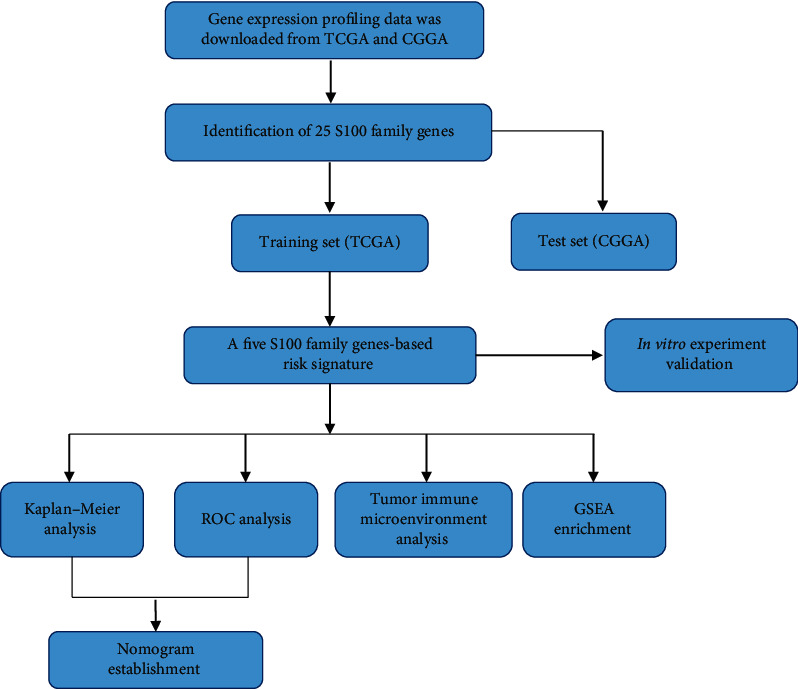
Flow chart of study design.

**Figure 2:**
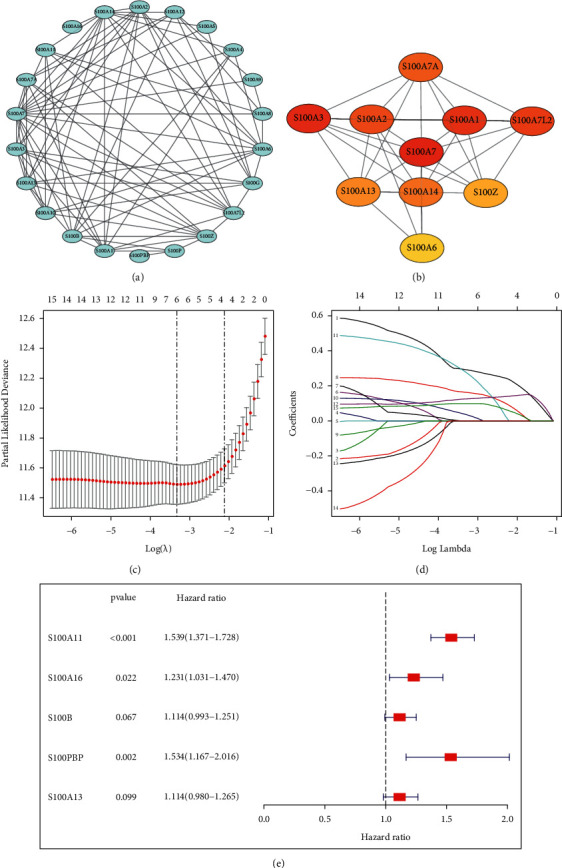
Establishment of a prognostic signature of glioma on the basis of S100 family genes. (a, b) Protein-protein interaction (PPI) among S100 family genes and the 10 hub genes were identified. (c, d) Multivariate Cox and LASSO regression analyses. (e) Forest plots of risk genes related to the prognosis prediction nomogram.

The flow chart of this study is shown in Figure 1. A total of 25 genes of S100 family were retrieved from the previous literature and TCGA/CGGA databases [6, 17]. To better explore the interaction among these genes, we established the PPI network comprising 22 nodes and 84 edges (Figure 2(a)). The 10 genes with the highest MCC score were identified by the STRING database and Cytoscape software (Figure 2(b)), suggesting their important role in human cancers.

To develop a specific S100 family-based signature for glioma prognosis, this study carried out univariate, multivariate, and LASSO regression based on the S100 family genes in the TCGA training data set. Finally, five S100 family genes (S100A11, S100A16, S100B, S100PBP, S100A13) were identified as key prognostic genes to establish the risk model (Figures 2(c)–2(e)). Risk score value was determined by this formula: risk score = (S100A11 × 0.431096) + (S100A16 × 0.208028) + (S100B × 0.108247) + (S100PBP × 0.427990) + (S100A13 × 0.107616).

**Figure 3:**
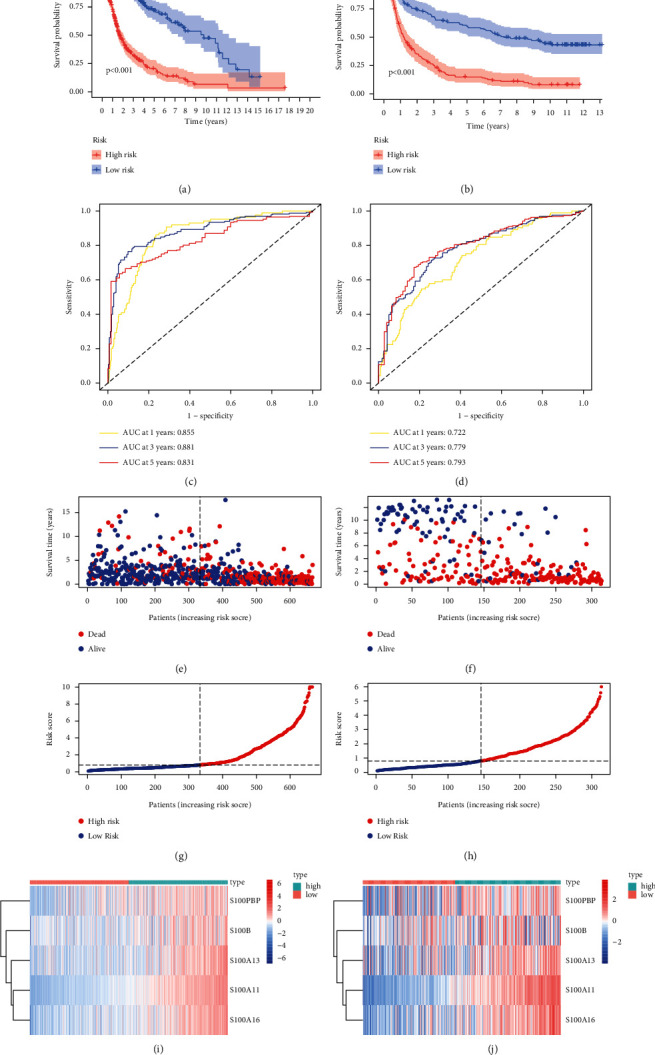
Identification of prognosis prediction performance of S100 family-based model of glioma from TCGA and CGGA databases. (a, b) The Kaplan–Meier (K-M) curve analysis for low- and high-risk patients based on risk score. (c, d) ROC analysis of 1-, 3-, and 5-year survival. (e, f) Patient survival status on the dot pot and survival time with different risk scores. (g, h) Patient distribution of groups and rank of risk score. (i, j) A heatmap of gene levels between high- and low-risk patients.

Patients were classified as a low- or high-risk group according to the median risk score value.  Figure 3 displays the prediction performance of our constructed nomogram in TCGA and CGGA databases. As revealed by Kaplan-Meier analysis, high-risk group had markedly short OS compared with low-risk group (*P* < 0.001; Figures 3(a) and 3(b)). We then used time-dependent ROC curves to determine the predictive reliability of this model, and the AUCs of ROC analyses are shown in Figures 3(c) and 3(d). Moreover, risk score values of glioma cases and the corresponding distribution are shown in Figures 3(e)–3(f), the survival status of each patient on the dot plot is marked in Figures 3(g)–3(h), and the heatmap of gene expression patterns is described in Figures 3(i)–3(f). The above findings suggested that our constructed prognosis prediction nomogram could be a good prognostic indicator in patients with glioma.

### 3.2. Independent Prognostic Value of the Five-S100 Family Gene Signature

**Figure 4:**
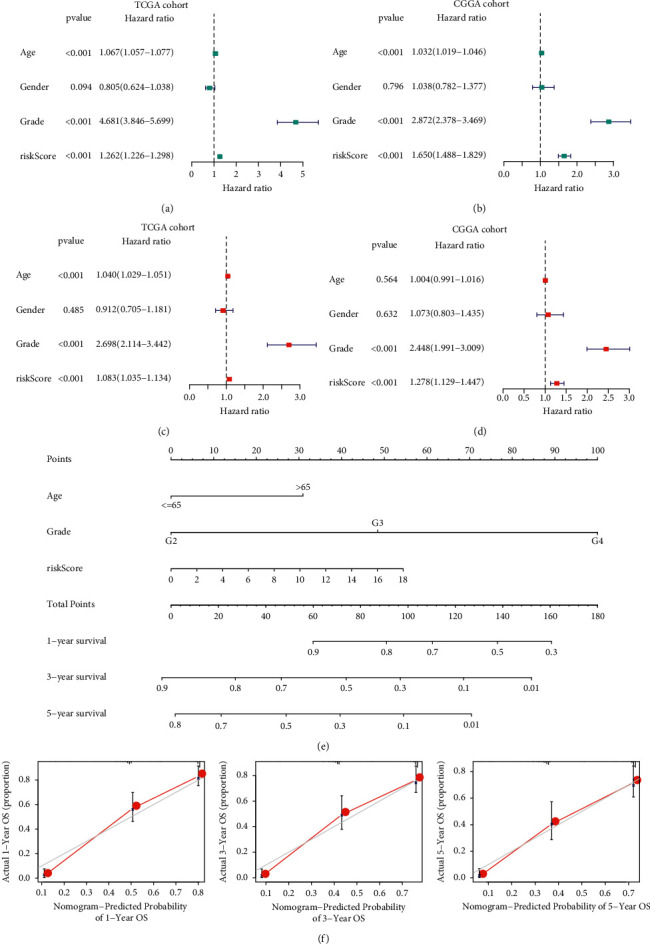
Independent prognosis significance of S100 family-based signature. (a–d) Univariate as well as multivariate Cox regression for OS in glioma cases from TCGA and CGGA databases. (e) A model constructed by incorporating clinical features and signature. (f) Calibration curve showing the nomogram prediction performance in 1-, 3-, and 5-year OS.

We performed univariate and multivariate Cox regression to determine whether the five-S100 family gene signature could be independent of other clinical parameters (age, gender, WHO grade, and risk score) as a predictor for patients with glioma. As revealed by univariate analysis, age, risk score value and grade were significantly related to patients' OS in both data sets (*P* < 0.001), only gender was not (Figures 4(a) and 4(c)). Upon multivariate Cox regression, age, risk score value and grade were the independent factors to predict OS for TCGA-derived patients (*P* < 0.001), whereas only grade and risk score remained statistically significant in the CGGA cohort (*P* < 0.001; Figures 4(b) and 4(d)). The above findings suggested that our constructed model served as an independent predictor for the prognosis of glioma patients.

### 3.3. Nomogram Analysis

This study constructed the nomogram based on risk score values and clinical features of patients for predicting their risk of survival using “rms” package in R software (Figure 4(e)). The nomogram integrated age, grade, and risk score, and each factor was employed for obtaining relevant score summary, as well as overall score for respective samples. The higher scores indicated a worse prognosis. In the calibration curve, the predictive and actual survival showed a good consistency in 1-, 3-, and 5-year OS (Figure 4(f)). The nomogram model passed the PH assumption and had no statistically significant deviation (*P* < 0.05) (Figure S4). In brief, this nomogram model performed well in predicting glioma survival.

### 3.4. GSEA Identifies S100 Family-Based Signature-Related Signaling Pathways

**Figure 5:**
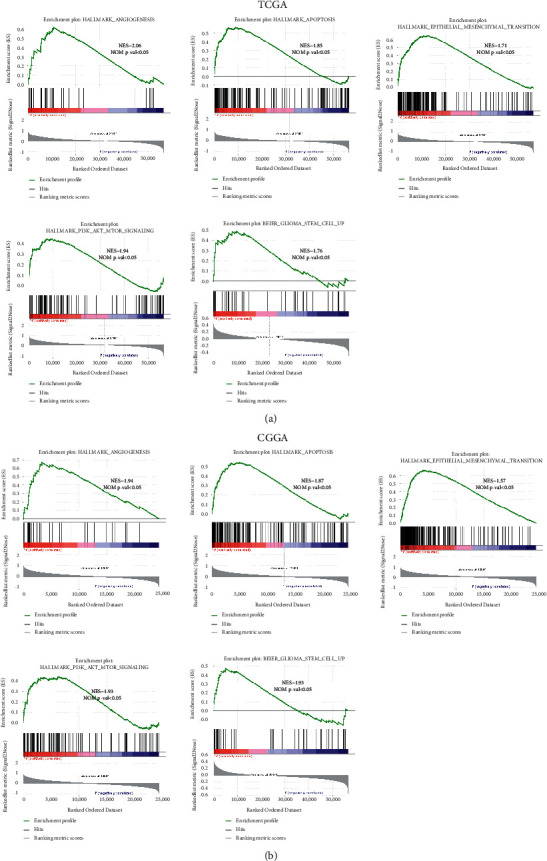
GSEA identifying the S100 family-based signature-related signaling pathways. (a) GSEA pathways were enriched into hallmarks of malignant tumors for TCGA-derived high-risk patients. (b) Results were verified using the CGGA database.

The constructed S100 family-based signature had potent stratification ability in the prediction of glioma OS, promoting us to investigate the related signal transduction pathways. GSEA was conducted to compare the gene sets enriched in low- and high-risk groups from both data sets. As a result, for TCGA-derived high-risk patients, GSEA pathways were associated with angiogenesis, apoptosis, PI3K-AKT-MTOR signal pathway, epithelial-mesenchymal transition, and glioma stem cell pathway (Figure 5(a)), which were verified using the CGGA database (Figure 5(b)).

### 3.5. Immune Landscape between Low- and High-Risk Glioma Patients

More and more studies indicate that tumor development is also affected by the tumor immune microenvironment (TIME). It is necessary to examine the impact of prediction nomogram on TIICs in glioma patients. CIBERSORT with the LM22 signature matrix was adopted for estimating the heterogeneities in 22 kinds of TIIC infiltrating levels in both groups.

**Figure 6:**
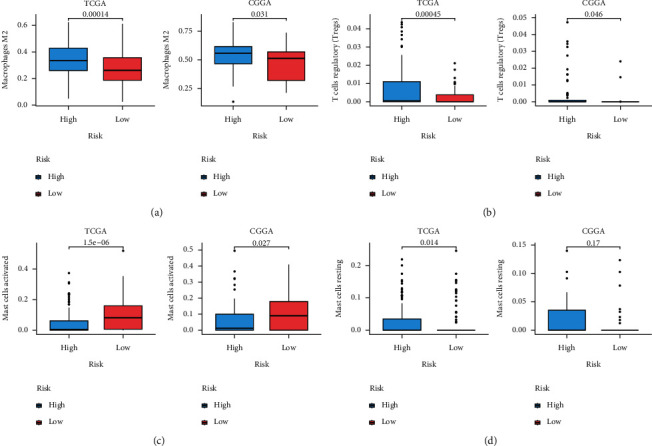
Association of risk score with TIIC infiltration. Box plots showing the TIICs with significant difference: (a) M2 macrophages, (b) Regulatory *T* cells, (c) activated Mast cells, and (d) resting Mast cells between low- and high-risk patients with glioma in TCGA and CGGA databases.

As shown in Figure 6, high-risk patients with glioma showed markedly increased M2 macrophage and Treg proportions, whereas apparently decreased activated Mast cell proportion in both CGGA and TCGA cohorts. Additionally, high-risk group had a markedly increased proportion of resting Mast cells compared with low-risk group in TCGA, but there was no significant difference in CGGA. Based on the Human Protein Atlas (HPA) database (http://www.proteinatlas.org), we also found that M2-related molecular (CD163) and Treg-related makers (CD25, STAT5B, and IL-10) were highly expressed in GBM tissues (Figure S1). The qRT-PCR results revealed that two important immune checkpoints, TGF-*β* and IL-10, were significantly higher in GBM cells than in NHA control (Figure S2).

### 3.6. Immune Checkpoint Inhibitors (ICIs) of S100 Family-Based Signature

**Figure 7:**
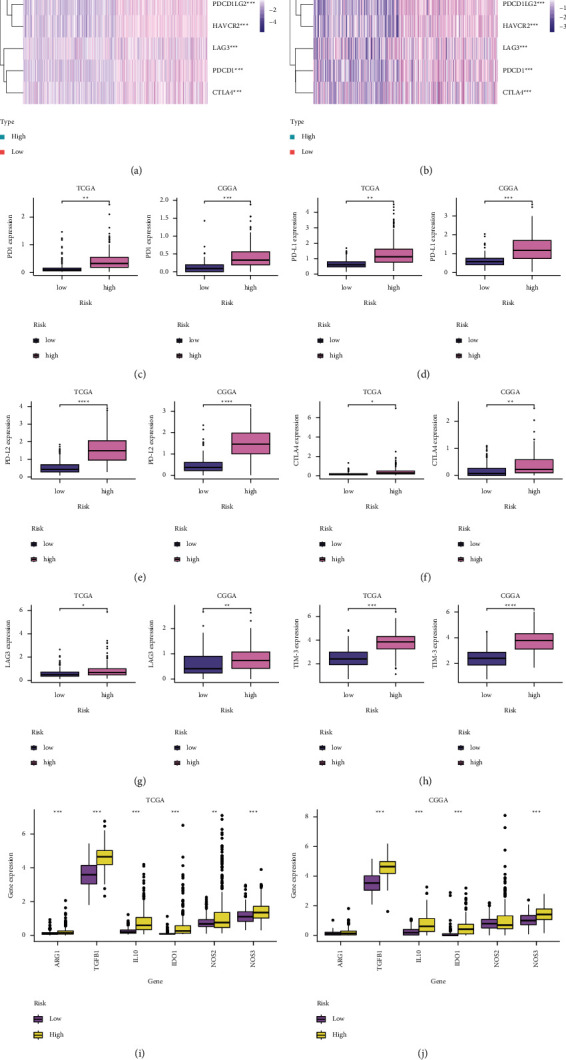
The estimation of two prognostic subtypes in immunosuppressive microenvironment. (a, b) Heatmap showing expressions of immune checkpoints between high- and low-risk patients derived from TCGA and CGGA databases. (c) PD-1 levels between high- and low-risk patients; (d) PD-L1 levels within high- and low-risk patients; (e) PD-L2 levels within low- and high-risk groups; (f) CTLA-4 expression in low- and high-risk patients; (g) LAG3 levels within low- and high-risk patients; (h) TIM-3 levels within low- and high-risk patients. (i, j) Cytokine levels in tumor immunosuppressive microenvironment between low- and high-risk patients. ^*∗*^*P* < 0.05, ^∗∗^*P* < 0.01, ^∗∗∗^*P* < 0.001, and ^∗∗∗∗^*P* < 0.0001.

ICIs have vital functions in maintaining immune homeostasis, which can be utilized by cancer cells to escape the immune response. Here, this study analyzed ICIs levels in both groups. As a result, high-risk patients had markedly increased levels of checkpoints like PD-1, PD-L1, PD-L2, TIM-3, LAG3, and CTLA-4 compared with low-group patients (Figures 7(a)–7(h)). Additionally, the levels of immunosuppressive cytokines (ARG1, TGF-*β*, IL-10, IDO1, MOS2, MOS3) increased among high-risk cases (Figures 7(i)–7(j)).

The above findings suggested that high-risk patients were more likely to develop the immunosuppressive microenvironment by upregulating immune checkpoints and immunosuppressive cytokines.

### 3.7. Verification of the Target Genes

**Figure 8:**
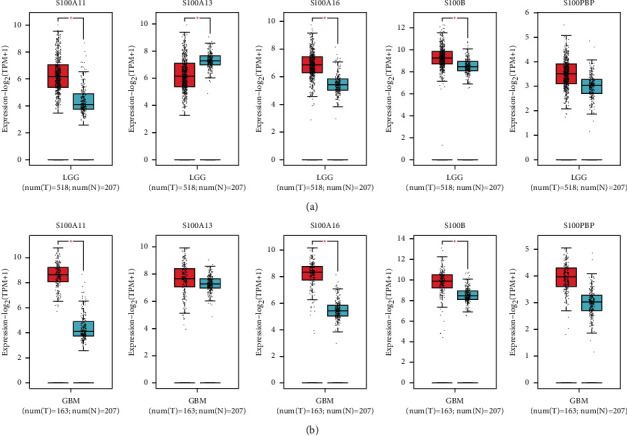
Expression of five signature-related genes based GEPIA database. (a) S100A11, S100A13, S100A16, S100B, and S100PBP levels between low-grade glioma (LGG) and normal tissues. (b) Gene levels within glioblastoma (GBM) and noncarcinoma samples.

**Figure 9:**
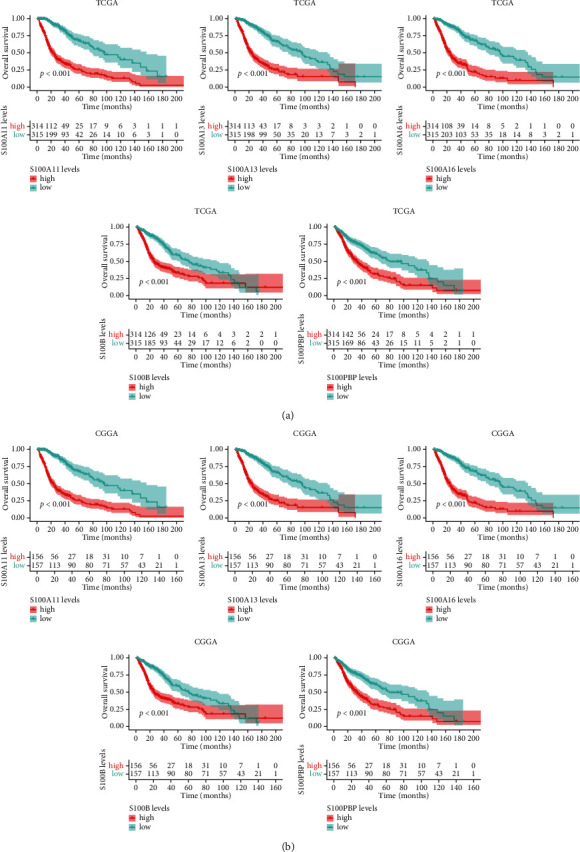
Prognosis of five signature-related genes based on TCGA and CGGA database. (a) Prognostic value of S100A11, S100A13, S100A16, S100B, and S100PBP in glioma from TCGA database. (b) Prognostic value of these genes in glioma from CGGA database.

Furthermore, the databases CGGA and GEPIA were adopted to verify the relationship between the expression of those 5 signature-related genes and patient survival. For the GEPIA-derived cohort, the expression levels of S100A11, S100A16, and S100B in low-grade glioma (LGG) and GBM samples increased compared with those in noncarcinoma samples, while S10013 in LGG tissue was upregulated in normal tissues (Figure 8). In TCGA and CGGA database, we performed a series of survival analyses to reveal the prognostic value of target genes of the signature in glioma patients. According to Figure 9, the group with high expression of S100A11, S100A13, S100A16, S100B, and S100PBP showed shorter OS relative to the group with low expression for all patients with glioma in the TCGA database (*P* < 0.001). In the CCGA database, the OS rate in patients with high levels of S10011, S100A16, and S100B was markedly worse than those with low expression (*P* < 0.001). Then, a subgroup survival analysis was also performed for patients with LGG and HGG (Figure S3). In general, upregulated target gene mRNA expression of the S100 family-based signature predicted dismal prognostic outcomes.

### 3.8. S100B Mediates GBM Cell Growth and Migration

**Figure 10:**
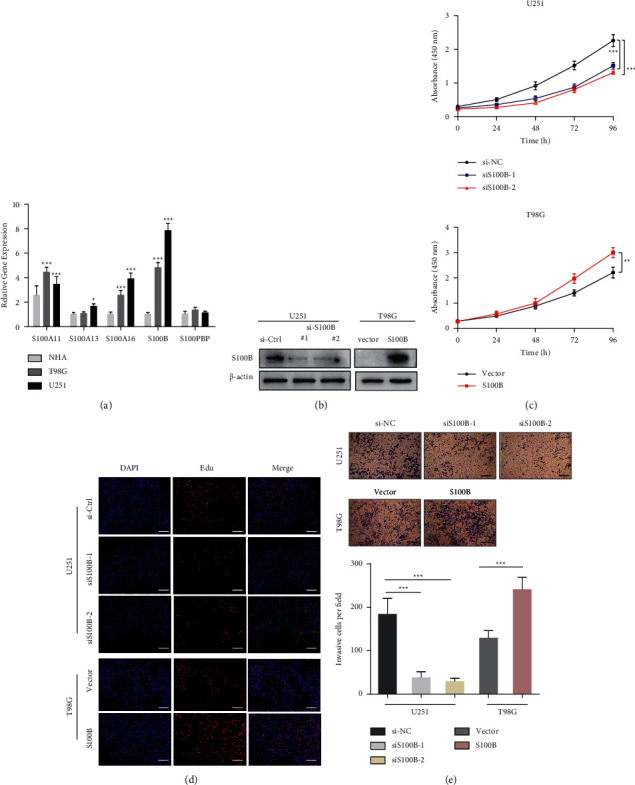
S100B mediates GBM cell proliferation and migration. (a) qPCR results showing 5 signature-related genes levels within GBM cells and NHA controls. (b) WB results revealing S100B was efficiently knocked down within U251 cells and overexpressed within T98G cells. (c) CCK-8 assay of U251 cells subjected to specific siRNA transfection and T98G cells after S100B plasmid or vector transfection. (d) EdU assay of U251 cells subjected to specific siRNA transfection and T98G cells S100B plasmid transfection. (e) Transwell assay of U251 cells subjected to specific siRNA transfection and T98G cells S100B plasmid transfection. ^*∗*^*P* < 0.05, ^∗∗^*P* < 0.01, and ^∗∗∗^*P* < 0.001.

To further validate this prognostic model, S100B gene was selected as a representative to carry out the functional experiments for the following reasons. Firstly, the S100A11, S100A16, and S100B expression levels were significantly upregulated in glioma tissues than in normal controls from GEPIA database. Secondly, the survival analysis further demonstrated the significant prognostic power of these 3 signature-related genes in TCGA and CGGA cohorts. Then, the qRT-PCR analysis showed that S100B expression was increased more markedly in GBM cell lines (Figure 10(a)), indicating that S100B may serve as an important prognostic biomarker.

S100B expression level was relatively higher in U251 cells than in T98G cells. Therefore, we knocked down the S100B expression in U251 cells by si-S100B transfection, and upregulated S100B in T98G cells (Figure 10(b)). This study conducted CCK-8 and EdU assays for detecting S100B's effect on the proliferation of GBM cells. As revealed by CCK-8 assay, downregulated S100B expression markedly inhibited U251 cell viability, with its overexpression in T98G cells and yielded the opposite effect (Figure 10(c)). According to EdU assay, inhibiting S100B dramatically decreased the percentage of EdU‐positive U251 cells, and overexpressing S100B increased EdU-positive T98G cells (Figure 10(d)). Also, this study conducted a transwell assay for investigating S100B's function in GBM migration and invasion. Silencing S100B expression by siRNA obviously decrease the number of invaded cells, whereas upregulating S100B resulted in more invaded cells (Figure 10(e)). The findings suggested that S100B promoted GBM cell growth and migration.

## 4. Discussion

Glioma is the most common type of brain tumor originating from neuroglial progenitor cells. Typically, the blood-brain barrier (BBB), comprising capillaries, basilar membranes, and endothelial cells, is a major reason for limiting the progress of antitumor drugs. With the recent advances in high-throughput technology, identifying novel prognostic markers and therapeutic targets may help improve glioma survival. Many S100 family proteins showed high expression levels within the nervous system [18]. We speculated that S100 family genes might exhibit a potent prognostic value for glioma patients.

A growing amount of open-sourced online platforms and genomic data have made it possible to explore family gene expression levels in glioma as well as the corresponding clinical significance. The present work analyzed S100 family genes and constructed a robust nomogram on this basis to predict glioma OS. Using Cox hazards and LASSO regression, five S100 family genes were identified for the prognostic model. The nomogram reliability, prediction performance, and stability were next analyzed and validated. As a result, the constructed signature could discriminate glioma prognosis with high accuracy. In addition, we created a nomogram consisting of clinical features and risk scores to present a personalized survival prediction for each patient with glioma. The calibration curves showed that the predicted patient survival was close to the actual measurement, indicating good predictive effects of the nomogram for survival time. Our signature, therefore, has great potential to be a clinical prognostic and predictive biomarker of glioma.

By focusing on the five signature-related genes of S100 family in this study, most of which have important functions in cancer genesis and development. S100A11, is also called calgizzarin or S100C, is localized both in the nucleus and in the cytoplasm. S100A11 binds to RAGE receptor thereby increasing epidermal growth factor (EGF) protein expression and stimulating cell growth [19]. It has been reported that S100A11 shows overexpression in many cancer types, including glioblastoma (GBM) [20–23]. S100A13 is identified to be involved in the nonclassical protein export, containing fibroblast growth factor (FGF), interleukin-1*α* (IL-1*α*), and synaptotagmins [24]. Growing evidence showed that S100A13 has a strong relationship with tumorigenesis [25–27], and it has been proved as a novel biomarker for papillary thyroid carcinomas (PTC) [28, 29]. Interestingly, S100A13 shows differential expression in brain development process, which suggests that it is of great importance to maintain the function of nervous system [30]. S100A16 is a recently discovered member of the S100 family obtained from astrocytoma, which is structurally more stable than other S100 genes [31]. S100A16 overexpression is detected in different cancers, including pancreas cancer, lung cancer, ovarian cancer, bladder cancer, and thyroid gland cancers [32]. S100B is a nervous system-specific protein that is mainly secreted from astrocytes. S100B is widely involved in the regulation of phosphorylation, protein degradation, cellular proliferation, and differentiation. Additionally, serum of S100B is used as the diagnostic biomarker for melanoma for a long time, which has also been adopted to be the candidate predicting factor for lung cancer brain metastasis recently [33, 34]. S100PBP is differentially expressed in various organs and disease states, which is dependent on tissue and cancer type. In breast cancer, S100PBP expression was markedly related to patient prognosis and different metastatic sites [35]. S100PBP level is suggested to be related to pancreatic ductal adenocarcinoma [36]. The biological roles of these five genes in cancer have partially provided clues for understanding the diagnostic and prognostic significance of the risk model in glioma. In our study, we demonstrated that most of these genes show high expression within GBM, and glioma patients with high expression levels have a shorter survival time than those with low expression. Moreover, we chose S100B as representative in subsequent functional analyses. The results showed that S100B expression markedly increased within GBM cells, and S100B promoted GBM cells growth, invasion, and migration. More investigations are needed to explore the molecular mechanisms underlying S100B and roles of other markers in the model in GBM.

Functional annotation of the S100 family-based signature via GSEA showed that there are a series of biological functions, such as PI3K-AKT-MTOR signaling, angiogenesis, apoptosis, epithelial-mesenchymal transition, and glioma stem cell pathways. It is worth mentioning here that apart from cancer-associated pathway, cancer stem cells (CSCs) are highly enriched in the high-risk group. CSCs are known as a rare population of self-renewing tumor cells, which contribute mainly to tumor recurrence and resistant to therapy [37, 38]. These findings indicated that high-risk patients based on the prognostic signature are more predisposed to tumorigenesis, recurrence, and resistance. In the future, more functional experiments are expected to explore the role of these five signature-related genes in glioma stem cells.

In addition, accumulating evidence suggests that TIME exerts an important part in glioma progress and development [39]. As a result, this study also examined the association of TIIC infiltration levels with risk score value in the prognosis mode. The high-risk group showed increased fractions of Treg cells and M2 macrophages phenotype. Macrophages can be divided into classically activated M1 macrophages and alternatively activated M2 macrophages. It is clear that M1 macrophages are involved in the antitumor immune response, and M2 macrophages are mainly responsible for tumor initiation, growth, and metastasis. It is also noted that Treg could promote tumor progression by specifically inhibiting tumor-reactive *T* cells [40]. Consistent with these, our study suggested that the increased tumor-associated M2 macrophages and Tregs are related to poor prognosis of glioma, probably due to their involvement in immune invasion.

Tumor immune cytokines and checkpoints are considered important factors to determine glioma prognosis and efficacy [41]. Interleukin-10 (IL-10) and transforming growth factor-*β* (TGF-*β*) represent two typical immunosuppressive cytokines within TIME. TGF-*β* is known to inhibit immune responses through suppressing the activity of NK-cells, regulating the generation of proinflammatory cytokines, and changing the differentiation of *T* cells [42]. IL-10 is an anti-inflammatory cytokine that is broadly expressed by various immune cells, including M2 macrophages, myeloid dendritic cells (DCs), Th1, Th2, and Treg cells. Especially, Treg-derived IL-10 can enhance Treg function and involve in Treg-induced immune regulation [43]. In our study, immunosuppressive cytokines, TGF-*β* and IL-10, were upregulated in the high-risk group. In addition, as the immune checkpoints are often used to escape immune surveillance by cancer cells, we also explore the response of checkpoint inhibitors (e.g., PD-1, PD-L1, PD-L2, CTLA-4, LAG3, and TIM-3) and discovered that many of these genes significantly increased in the high-risk group. Based on our results, high-risk glioma patients may have a better response for immunotherapy.

However, there are some limitations in the present study. Firstly, the data downloaded from public sources was restricted and incomplete, as well as no clinical samples were used for validation. Secondly, the five genes in the signature required more in vitro and in vivo experiments to verify their function in glioma. Finally, further researches in multicenter, large-scale, and prospective clinical trials are needed to confirm the risk model's predictive efficacy.

## 5. Conclusion

This work first constructed and validated an S100 family-based signature for the prognosis of glioma. This risk signature can be used as a factor to independently predict the glioma patients' OS. We also proved an important value of this model in glioma immune microenvironment. Moreover, we identified that S100B, as an important biomarker, could promote GBM cell growth and invasion in vitro. Our study provided a prognostic model and promising biomarkers for glioma diagnosis and treatment.

## Data Availability

All data sets used in the present work are included within this manuscript. These data are available in TCGA (https://portal.gdc.cancer.gov/) and CGGA (http://www.cgga.org.cn) databases.

## Supplementary Materials

Supplementary Materials Figure S1: representative IHC staining for makers relating to M2 macrophages (CD163) and Treg cells (CD25, STAT5B, and IL-10) in normal and tumor tissues from the Human Protein Atlas. Figure S2: qPCR analysis of TGF-*β* and IL-10 expression in NHA and glioma cell lines. Figure S3: subgroup analysis for prognosis of five genes incorporated into the signature. Figure S4: plot of the *p* values of the global PH assumption tests. Table S1: primer sequences used in this study ().
