# Supplementary material for: A Novel S100 Family-Based Signature Associated with Prognosis and Immune Microenvironment in Glioma
Source: J Oncol. 2021 Sep 29;2021:3586589. doi: 10.1155/2021/3586589 (PMC8548170; doi:10.1155/2021/3586589)
Supplement: Supplementary Materials — Figure S1: representative IHC staining for makers relating to M2 macrophages (CD163) and Treg cells (CD25, STAT5B, and IL-10) in normal and tumor tissues from the Human Protein Atlas. Figure S2: qPCR analysis of TGF-β and IL-10 expression in NHA and glioma cell lines. Figure S3: subgroup analysis for prognosis of five genes incorporated into the signature. Figure S4: plot of the p values of the global PH assumption tests. Table S1: primer sequences used in this study (). [file 3586589.f1.docx]

**A novel S100 family-based signature Associated with Prognosis and Immune microenvironment in** **Glioma**

**Yifang Hu^1,†^, Jiahang Song^2,†^, Zhen Wang^3,†^, Jingbao Kan^1^, Yaoqi Ge^1^, Dan Wang^1^, Rihua Zhang^1^, Wensong Zhang^4,*^, Yun Liu^1,5,*^**

^1^ Department of Geriatrics, The First Affiliated Hospital of Nanjing Medical University, Nanjing, Jiangsu, China

^2^ Department of Radiation Oncology, The First Affiliated Hospital of Nanjing Medical University, Nanjing, Jiangsu, China

^3^ Department of Neurosurgery, The Affiliated Brain Hospital with Nanjing Medical University, Fourth Clinical College of Nanjing Medical University, Nanjing, Jiangsu, China

^4^ Department of Pharmacy, The First Affiliated Hospital of Nanjing Medical University, Nanjing, Jiangsu, China

^5^ Department of Medical Informatics, School of Biomedical Engineering and Informatics, Nanjing Medical University, Nanjing, Jiangsu, China

*** Correspondence:**
Yun Liu
[liuyun@njmu.edu.cn](mailto:liuyun@njmu.edu.cn)

Wensong Zhang

[zhangwensong91@126.com](mailto:zhangwensong91@126.com)

†These authors have contributed equally to this work and share first authorship.

Supplemental Tables, Figures and Data

**Figure S1**


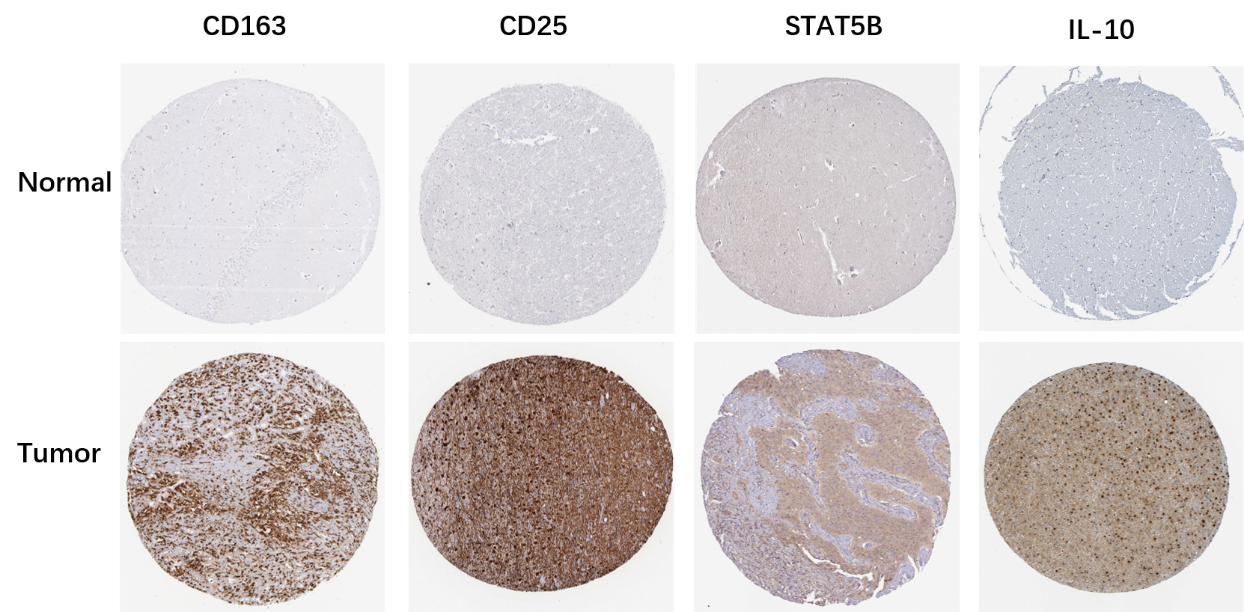
**Figure S1: Representative IHC staining for makers relating to M2 macrophages (CD163) and Treg cells (CD25, STAT5B and IL-10) in normal and tumor tissues from the Human Protein Atlas.**

**Figure S2**


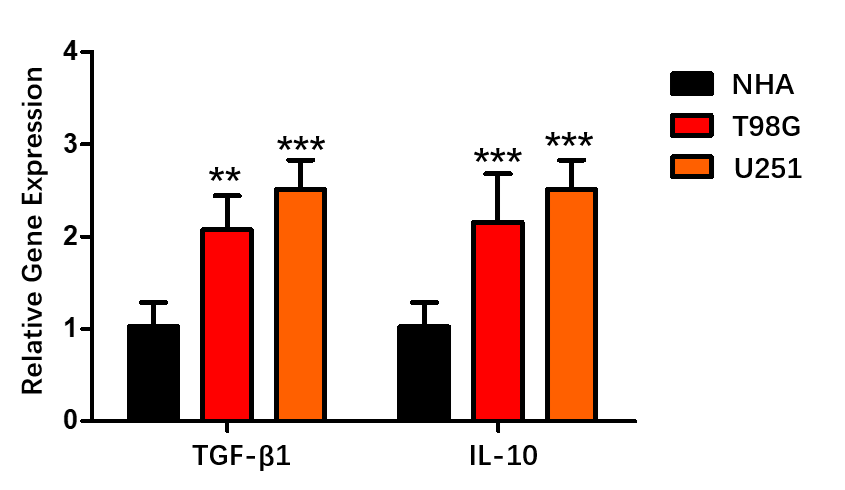


**Figure S2: qPCR analysis of *TGF-β* and *IL-10* expression in NHA and glioma cell lines.**

**Figure S3**


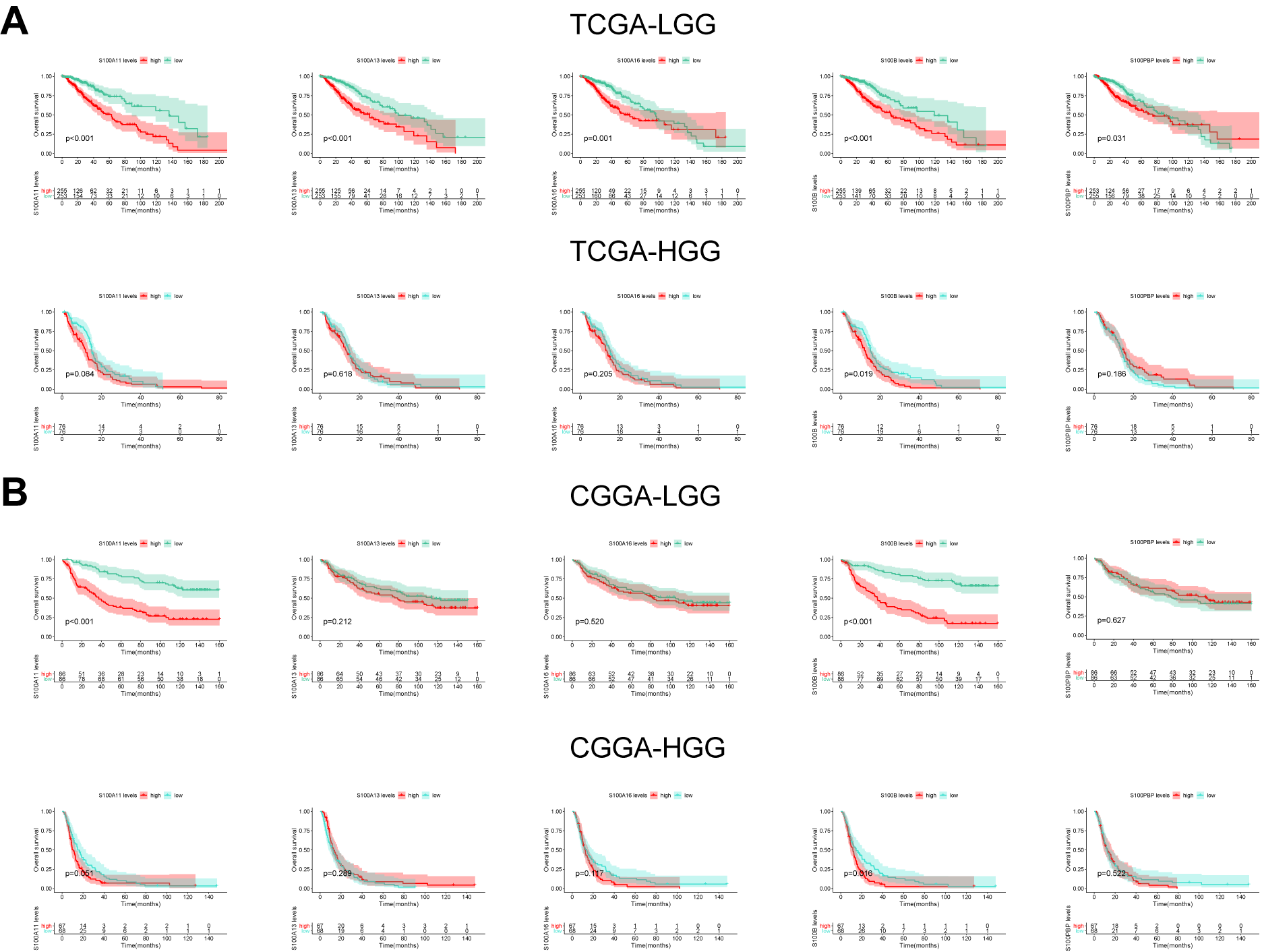


**Figure S3: Subgroup analysis for prognosis of five genes incorporated into the signature**. (**A**) Prognostic value of *S100A11, S100A13, S100A16, S100B* and *S100PBP* in LGG and GBM patients from TCGA database. (**B**) Prognostic value of these genes in in LGG and HGG from CGGA database.

**Figure S4**


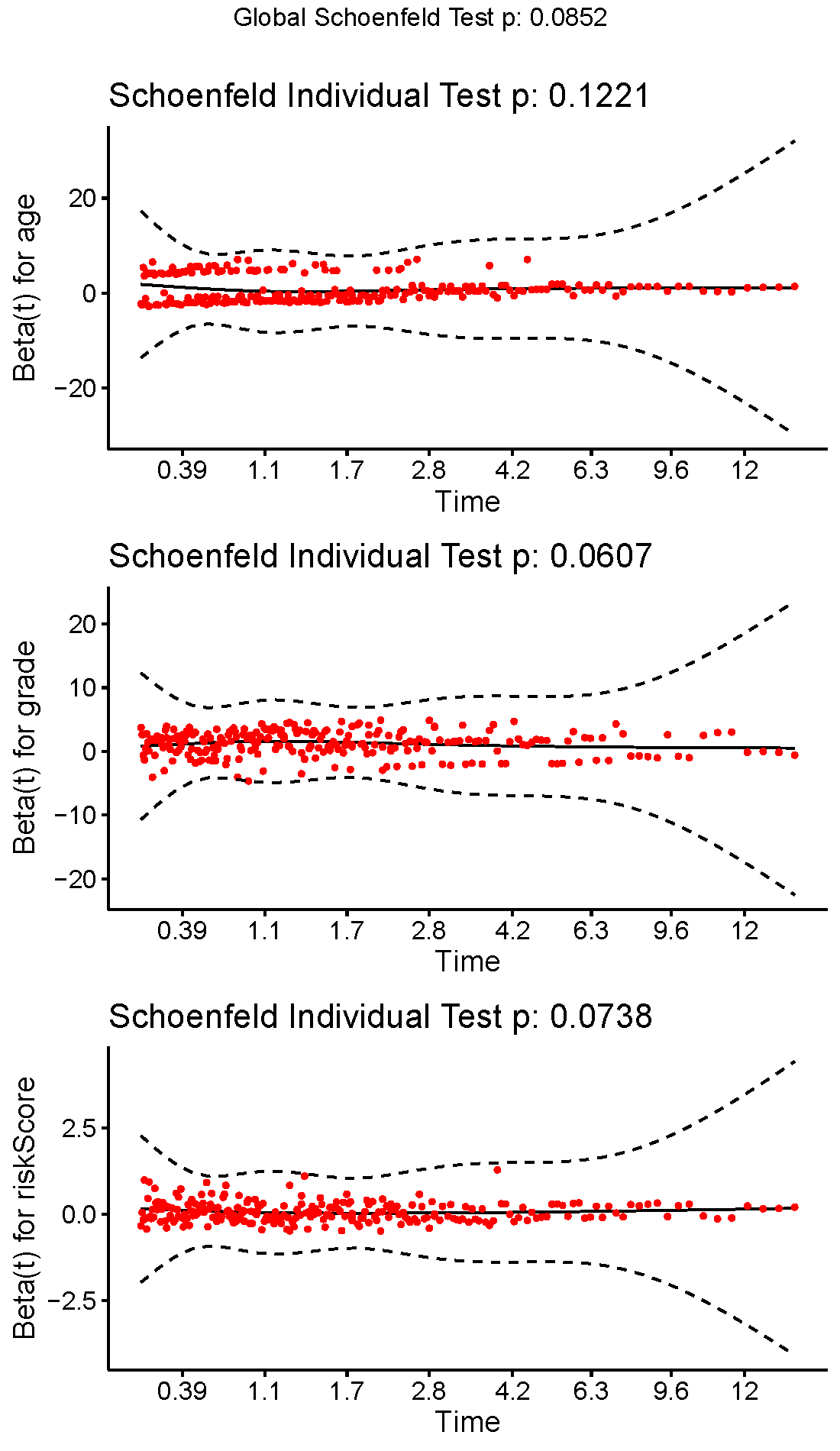


**Figure S4: Plot of the p-values of the global PH assumption tests.**

**Table S1**

**Primer sequences used in this study**

| **Primer set** | **Primers** | **Sequence (5’-3’)** | **Product size (bp)** |
| --- | --- | --- | --- |
| *S100B* | F | 5'- TGGCCCTCATCGACGTTTTC -3' | 248 |
|  | R | 5'- ATGTTCAAAGAACTCGTGGCA -3' |  |
| *S100A11* | F | 5'- ATGGCAAAAATCTCCAGCCCT -3' | 153 |
|  | R | 5'- TGTGAAGGCAGCTAGTTCTGTA -3' |  |
| *S100A13* | F | 5'- GATAGCCTCAGCGTCAACGAG -3' | 112 |
|  | R | 5'- CCTGATTCACATCCAAGCTCTT -3' |  |
| *S100A16* | F | 5'- AGTACAGCCTGGTCAAGAACA -3' | 90 |
|  | R | 5'- TCCCTGTGTCCGACAGCAT-3' |  |
| *S100PBP* | F | 5'- CCTGACAGTGAGAACCCTACG -3' | 108 |
|  | R | 5'- ACAACTTGACTTGTGTGAACCAC -3' |  |
| *β-Actin* | F | 5'- CATGTACGTTGCTATCCAGGC -3' | 152 |
|  | R | 5'- CTCCTTAATGTCACGCACGAT -3' |  |
| *TGF-β1* | F | 5'- CTAATGGTGGAAACCCACAACG -3' | 209 |
|  | R | 5'- TATCGCCAGGAATTGTTGCTG -3' |  |
| *IL-10* | F | 5'- GACTTTAAGGGTTACCTGGGTTG -3' | 112 |
|  | R | 5'- TCACATGCGCCTTGATGTCTG -3' |  |
